# Lymphocyte HVEM/BTLA co-expression after critical illness demonstrates severity indiscriminate upregulation, impacting critical illness-induced immunosuppression

**DOI:** 10.3389/fmed.2023.1176602

**Published:** 2023-05-25

**Authors:** Michelle E. Wakeley, Brandon E. Armstead, Chyna C. Gray, Elizabeth W. Tindal, Daithi S. Heffernan, Chun-Shiang Chung, Alfred Ayala

**Affiliations:** ^1^Division of Surgical Research, Department of Surgery, Rhode Island Hospital, Brown University, Providence, RI, United States; ^2^Graduate Pathobiology Program, Brown University, Providence, RI, United States; ^3^Molecular, Cellular and Developmental Biology Graduate Program, Brown University, Providence, RI, United States

**Keywords:** HVEM, BTLA, trauma, sepsis, immune dysfunction

## Abstract

**Introduction:**

The co-regulatory molecule, HVEM, can stimulate or inhibit immune function, but when co-expressed with BTLA, forms an inert complex preventing signaling. Altered HVEM or BTLA expression, separately have been associated with increased nosocomial infections in critical illness. Given that severe injury induces immunosuppression, we hypothesized that varying severity of shock and sepsis in murine models and critically ill patients would induce variable increases in HVEM/BTLA leukocyte co-expression.

**Methods:**

In this study, varying severities of murine models of critical illness were utilized to explore HVEM^+^BTLA^+^ co-expression in the thymic and splenic immune compartments, while circulating blood lymphocytes from critically ill patients were also assessed for HVEM^+^BTLA^+^ co-expression.

**Results:**

Higher severity murine models resulted in minimal change in HVEM^+^BTLA^+^ co-expression, while the lower severity model demonstrated increased HVEM^+^BTLA^+^ co-expression on thymic and splenic CD4^+^ lymphocytes and splenic B220^+^ lymphocytes at the 48-hour time point. Patients demonstrated increased co-expression of HVEM^+^BTLA^+^ on CD3^+^ lymphocytes compared to controls, as well as CD3^+^Ki67^-^ lymphocytes. Both L-CLP 48hr mice and critically ill patients demonstrated significant increases in TNF-α.

**Discussion:**

While HVEM increased on leukocytes after critical illness in mice and patients, changes in co-expression did not relate to degree of injury severity of murine model. Rather, co-expression increases were seen at later time points in lower severity models, suggesting this mechanism evolves temporally. Increased co-expression on CD3^+^ lymphocytes in patients on non-proliferating cells, and associated TNF-α level increases, suggest post-critical illness co-expression does associate with developing immune suppression.

## 1. Introduction

Immune suppression following critical illness, both after sepsis and following major traumatic injury, represents a well-documented but poorly understood phenomenon-lacking direct targeted therapies. Delayed mortality after critical illness is largely associated with the development of secondary infection and associated end-organ dysfunction (1, 2). Critical illness induces significant immune deficiency associated with secondary infection, long-term organ dysfunction, and increased risk of death. Patients who develop secondary infections fare worse than their counterparts when matched for injury severity and age (3, 4). Indicators for the risk of development of secondary infections include high levels of T lymphocyte apoptosis and a variety of genomic signatures that have been identified; however, no universally successful preventative treatments have been developed (4–6). Co-regulatory molecules, also referred to as checkpoint regulators, work to inform the immune system of the most appropriate response to an antigenic challenge, and as such are central mediators of this immune dysfunction (7, 8). PD-1 and CTLA-4 are well-characterized examples of co-regulatory molecules and, along with other similar co-regulatory molecules, are central mediators of this immune dysfunction (9).

Herpes Virus Entry Mediator (HVEM) is a TNF family transmembrane receptor expressed diffusely on solid organs and immune cells and is a coregulatory molecule, like PD-1 or CTLA-4 (10, 11). HVEM serves as a bidirectional switch stimulating or inhibiting immune function based on environmental cues. HVEM is expressed on T-cells, B-cells, NK cells, DCs, and myeloid cells, with high levels of expression on human naïve and memory B-cells, and constitutive expression on T cells (12). HVEMs interact with a variety of ligands including B and T lymphocyte attenuator protein, or BTLA, CD160, LIGHT, and Lymphotoxin alpha (LTα) (13). Generally, binding with any of these ligands occurs when HVEM is expressed in its *Trans* confirmation on the membrane. The ultimate signal is dependent on which ligand it binds and its downstream signaling complex, resulting in either stimulatory or inhibitory activity (11, 14, 15). This bidirectionality allows HVEM to orchestrate a diverse series of responses to environmental stimuli (16). When stimulated, HVEM signaling leads to activation of the RelA subunit of NFκB which results in downstream activation of inflammatory cytokines including TNF-α, IL-1β, IL-6, and IL-12p40 (17).

Adding to its complexity, HVEM has been shown to exist in *Cis*-confirmation on the cell membrane, and in these settings, it has been observed to be co-expressed with ligand BTLA (18). When co-expressed, HVEM and BTLA bind, forming an inert complex preventing all further downstream HVEM signaling in response to stimuli (18). This HVEM/BTLA complex competitively inhibits HVEM activation by ligands expressed in the surrounding microenvironment, ultimately helping maintain T cells in the naive state regardless of environmental cues for activation. LIGHT has been shown to bind HVEM while it is co-expressed with BLTA, but no stimulatory effect is generated, while other ligands such as BTLA on neighboring cells, lymphotoxin alpha, and CD160 are not able to bind to the complex at all (18).

Prior study has explored the expression of both HVEM and BTLA individually in murine models of critical illness, as well as in patients after both sepsis and trauma. Murine adults subjected to a model of indirect acute lung injury generated by hemorrhage followed by cecal ligation and puncture demonstrated that HVEM expression was upregulated in lung tissue, and this expression could successfully be knocked down by intra-tracheal administration of HVEM silencing RNA (siRNA). This HVEM siRNA administration resulted in a transient early survival benefit and preservation of lung tissue architecture, suggesting a role for HVEM blockade after trauma in protecting patients from indirect acute lung injury (19). HVEM signaling has been shown to mediate host immunity at mucosal barriers in mice, and the absence of the ability to signal via the HVEM pathway increases septic mortality in both gastrointestinal and respiratory infection murine models (12, 20–22).

BTLA expression has been shown to increase in CD4^+^ lymphocytes, monocytes, and granulocytes of septic ICU patients, with similar HVEM upregulation after sepsis (23). Furthermore, it has been shown that non-septic critically ill patients with greater than 80% BTLA expression on CD4^+^ T lymphocytes were at increased risk of developing secondary infection (24). In humans, HVEM expression has previously been shown to increase on CD3^+^ T-lymphocytes and granulocytes in the setting of sepsis (23, 25). Finally, in an investigation of HVEM expression on T-lymphocytes in a cohort of 30 trauma ICU patients, patients demonstrated lymphocyte loss and a significant increase in HVEM^+^ expression on CD3^+^ lymphocytes after trauma, with altered post-traumatic HVEM expression associated with increased risk of developing secondary infections (26). Furthermore, it was recently demonstrated that patients developing secondary infections have less circulating HVEM^+^CD3^+^ cells, implying that HVEM signaling in lymphocytes plays a role in maintaining host defense against infection after trauma (26).

Given the well-documented phenomenon of immunosuppression after severe critical illness and previously demonstrated expression changes in HVEM and BTLA in critically ill patients’ blood leukocytes, we hypothesize that mice subjected to sequential shock and sepsis (Hemorrhage [Hem]; cecal ligation; and puncture [CLP]) will show increased HVEM/BTLA immune cell co-expression when compared to mice subjected to CLP alone. Furthermore, we hypothesize that critically ill patients will similarly demonstrate increased HVEM^+^BTLA^+^ co-expression relative to the degree of injury, and this will impact immune cell behaviors.

## 2. Materials and methods

### 2.1. Mice

C57BL/6 male mice, aged 8–12 weeks, were purchased from the Jackson Laboratory (Bar Harbor, Maine) and housed in Rhode Island Hospital’s Central Research Facilities for 7 days before use. All protocols were conducted according to the National Institutes of Health guide for animal use and care and were approved by the Lifespan-Rhode Island Hospital Institutional animal care and use committee (approval numbers: 0054–18 and 5,054–21). Animals were fed standard mouse chow *ad libitum*, housed in ventilated racks, and kept under standard environmental conditions (12-h:12-h light/dark cycle, 68–72°F, and 30–70% humidity).

### 2.2. Animal models

Male mice were divided into a total of three treatment groups of varying severity with appropriate sham controls. These treatments included a model of indirect acute lung injury involving hemorrhage (Hem) followed by cecal ligation and puncture (CLP), a highly lethal severe CLP model, and a less severe CLP model (27, 28).

#### 2.2.1. Indirect acute lung injury (Hem/CLP) model

Our murine model of indirect acute lung injury (Hem/CLP, *N* = 11) was induced by hemorrhage (Hem) followed 24 h later by CLP. Anesthetized male mice underwent femoral artery cannulation and, subsequently, 30–40% of the blood volume was removed. Hypotension, defined as MAP 35 ± 5 mmHg, was induced and maintained for 90 min. Resuscitation was then provided with Lactated Ringers in a 4:1 ratio to the blood volume removed. Twenty-four hours after Hem, mice were then subject to CLP as described above in the L-CLP section. The sham group was anesthetized and underwent bilateral femoral artery cutdowns with suture ligation without any hemorrhage, followed 24 h later by sham CLP as described above in the Sham L-CLP section (Sham Hem/CLP, *N* = 11).

#### 2.2.2. Severe cecal ligation and puncture model

For the severe CLP (S-CLP) model, anesthetized male mice were subject to a midline laparotomy, followed by ligation of the entire cecum, followed by three puncture sites with a 22-gauge needle and subsequent extrusion of the cecal contents into the intraperitoneal space. Following abdominal closure with sterile suture, the mice were also treated with intramuscular lidocaine and 1 mL of subcutaneous Lactated Ringers solution, then euthanized at 24 h for tissue collection (S-CLP, *N* = 10). Sham animals were subject to anesthesia, laparotomy, and cecal manipulation without ligation or puncture, and then underwent abdominal closure and resuscitation before being euthanized at 24 h post-intervention (Sham S-CLP, *N* = 10).

#### 2.2.3. Less severe cecal ligation and puncture model

The less severe CLP (L-CLP) mice were treated with a standard CLP model as previously described, utilizing a midline laparotomy on anesthetized male mice, followed by ligation of the cecum approximately 1 cm from the cecal tip, three puncture sites with a 22-gauge needle, and subsequent extrusion of the cecal contents into the abdomen (29, 30). The laparotomy was closed with a sterile suture, and the animals were treated with intramuscular lidocaine and 1 mL of subcutaneous Lactated Ringer’s solution. Mice were subsequently euthanized at 24 h (24 h L-CLP, *N* = 6) or 48 h (48 h L-CLP, *N* = 6), with tissues harvested for subsequent studies. Control samples were subjected to sham CLP consisting of anesthesia, laparotomy, and cecal manipulation without ligation or puncture, and abdominal closure and resuscitation, then euthanized at 24 h post-procedure with tissues collected for study (Sham L-CLP, *N* = 7).

### 2.3. Patients

Critically ill adult patients (*N* = 23), age 18 years and older, were prospectively enrolled from the Surgical and Trauma Intensive Care Units (ICUs) at a single level 1 tertiary care center following appropriate informed consent under the Lifespan-RI Hospital Institutional Review Board approved protocol (approval number: 413013). Patients were included in the study if they had a trauma or sepsis-related critical illness requiring ICU admission and central venous catheter placement. All patients were enrolled within 24 h of ICU admission for either septic shock or severe traumatic injury. Peripheral blood samples were drawn within 48 h of ICU admission in patients presenting after traumatic injury, and within 48 h of demonstration of sepsis as scored by SIRS criteria (31). Patients were excluded if they required massive transfusion protocol activation and if death occurred within 48 h of ICU admission, pregnancy, and any patient with a documented history of leukemia or lymphoma. Age and sex-matched healthy volunteers were similarly enrolled, with peripheral blood samples collected on the day of enrollment (*N* = 14).

Patient charts were reviewed for clinical information including demographics, comorbidities, traumatic injuries sustained, and source of septic insult. Clinical data including vital signs, laboratory values, and physical exam findings were reviewed to calculate Acute Physiology of Chronic Health Evaluation II (APACHE II score) (Table 1). Each patient’s subsequent ICU and hospital course was reviewed for all complications, including the development of subsequent infections. Determination of infection, as well as all clinical care, was at the discretion of the treating team.

**Table 1:** Patient demographics.

	Healthy controls	Patients	Value of *p*
Number	14	23	–
Age	39.7 ± 17	56.4 ± 17.7	**0.007**
Male gender	7 (50%)	18 (76%)	0.07
WBC	–	14.1 ± 8.3 × 10^6^/ml	–
Active infection	–	14 (60.9%)	–
Traumatic injury	–	13 (56.5%)	–
APACHE II score	–	19.1 ± 6.9	–
Mortality	–	4 (17.4%)	–
Active infection	–	14 (60.9%)	–

Bolded values, indicate where a statistical significant difference of *p* < 0.05 was detected.

### 2.4. Flow cytometry

#### 2.4.1. Murine samples

**Figure 1:**
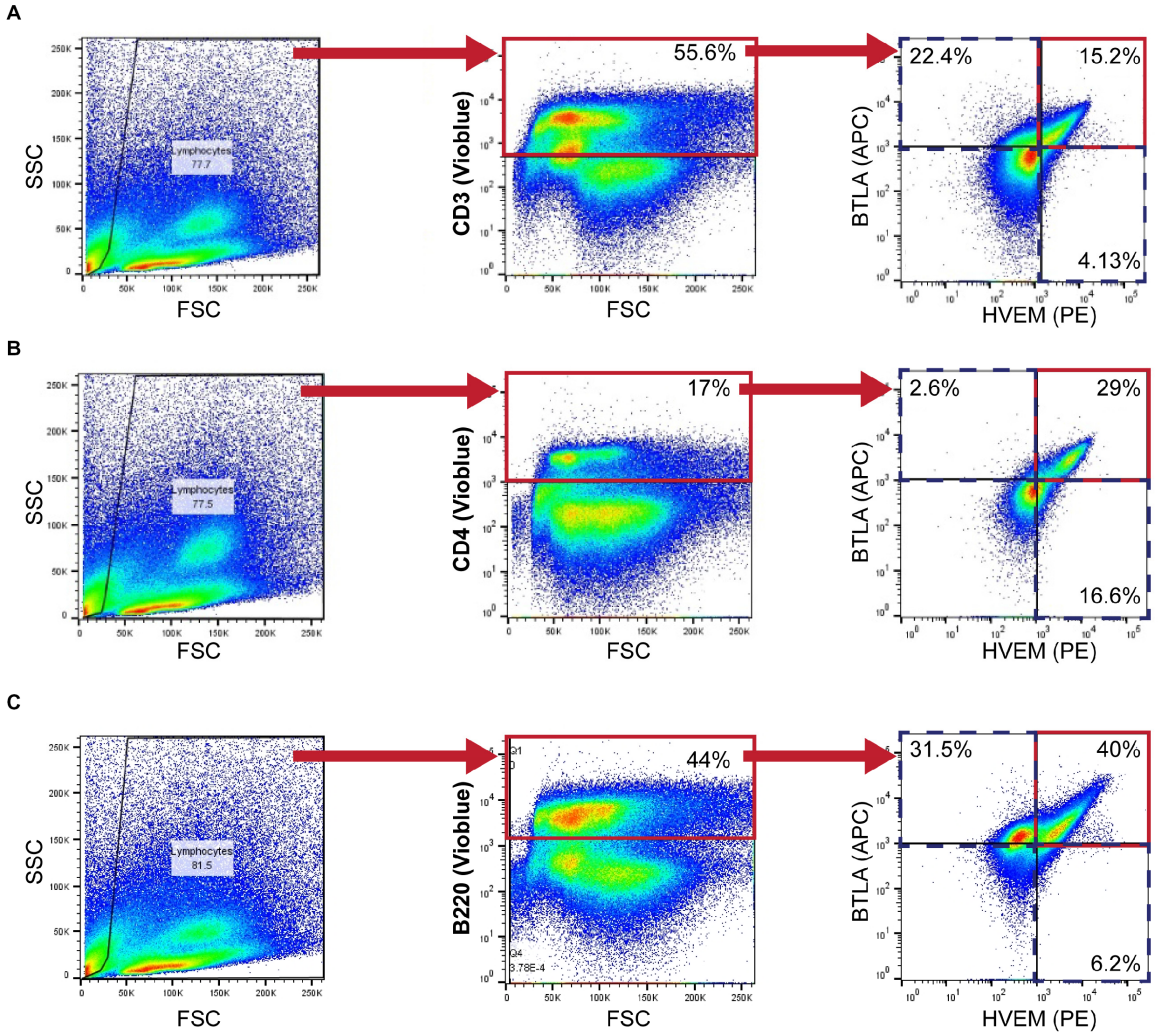
Murine flow cytometry gating strategy demonstrating CD3^+^ **(A)**, CD4^+^ **(B)**, and B220^+^ **(C)** gating for HVEM^+^ and BTLA^+^ co-expression demonstrated in a 24-h L-CLP spleen sample.

Mice were euthanized at 24 h following Hem/CLP and S-CLP procedures, and at either 24 or 48 h following the L-CLP procedure. Thymus and spleen tissues were harvested from mice and homogenized using frosted slides. Red blood cells were lysed using a Na^+^Cl^−^ gradient. Single-cell suspensions were stained with Trypan blue and counted using a hemacytometer at 10X magnification. Cells were diluted to a concentration of 10^6^ cells/mL in FACS buffer (2 mM EDTA, 0.5% BSA, 1X PBS). The following mAb conjugated to fluorochromes were used: CD3e-VioBlue (Miltenyi Biotec, Cat# 130–118-849; CD3^+^ T-cells), CD4-VioBlue (Miltenyi Biotec, Cat# 130–102-774; CD4^+^ T-cells), CD8a-VioBlue (Miltenyi Biotec, Cat# 130–102-431; CD8^+^ T-cells), CD45R (B220)-VioBlue (Miltenyi Biotec, Cat# 130–110-851; B220^+^), PE anti-mouse CD270 (BioLegend, San Diego, CA, United States, Cat# 136304; HVEM^+^), and APC-CD272 (BioLegend, Cat# 139110; BTLA^+^). Miltenyi MACSQuant was used to assess fluorescence. Data were analyzed with FlowJo version 9.3.2. Gating was established with a fluorescence minus one approach. Isotype control specimens were used to assess non-specific binding. Gating for each cell type explored is demonstrated in Figure 1.

Blood was collected in heparinized syringes at the time of euthanasia via direct cardiac puncture. Plasma was subsequently isolated from whole blood samples via centrifugation at 10,000 rpm, and supernatant (plasma) was collected and stored at-80°C.

#### 2.4.2. Human samples

**Figure 2:**
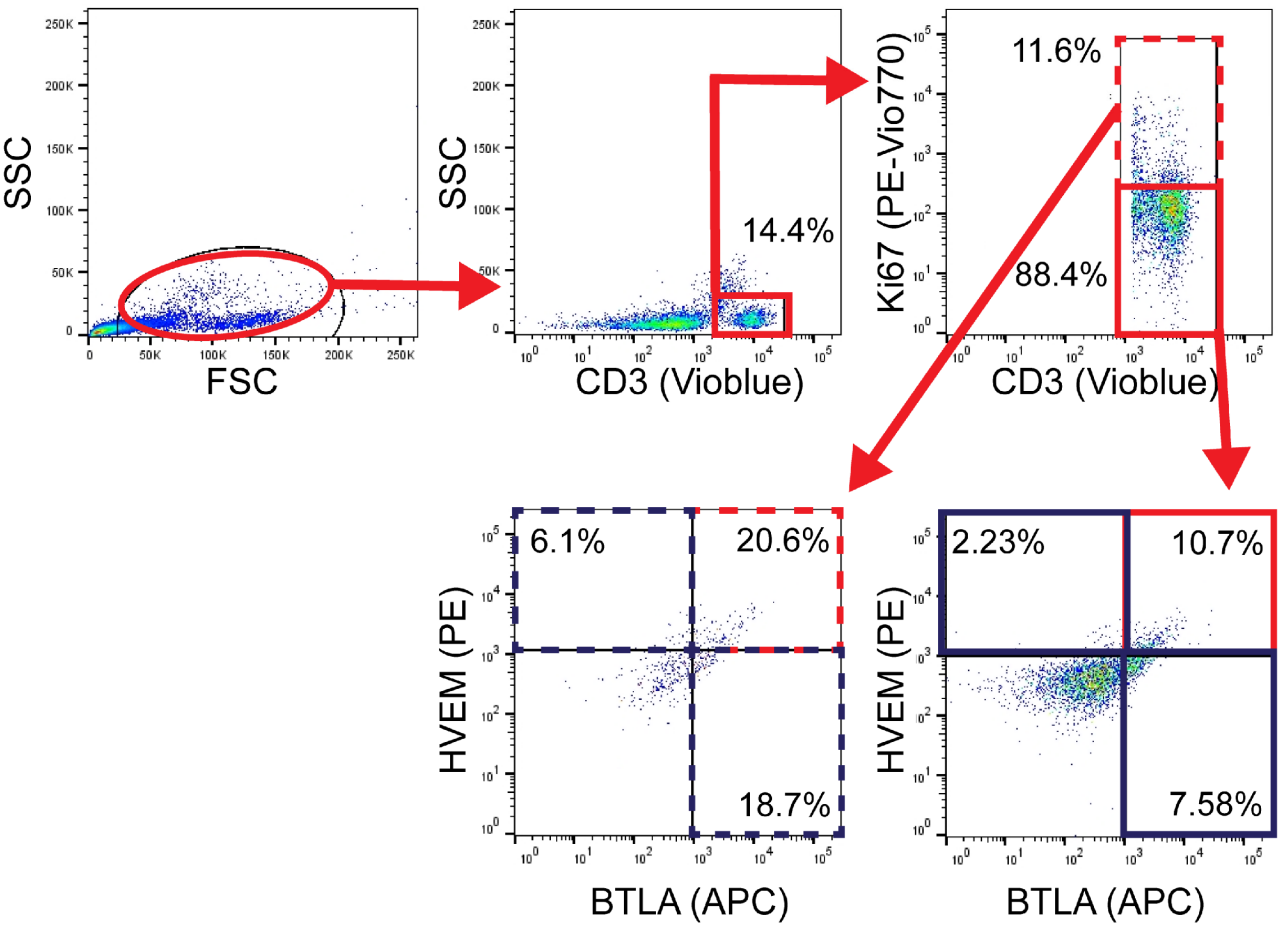
Patient flow gating strategy demonstrating CD3^+^ cells with Ki67^+^ expression, then subset evaluation of HVEM^+^ and BTLA^+^ expression in the Ki67^+^ and Ki67^−^ components, with gates set using fluorescence minus one technique.

Leukocytes were isolated from peripheral blood samples with histopaque separation and immediately analyzed fresh by flow cytometry as described above. The following mAb conjugated to fluorochromes were used: CD3-VioBlue, Clone BW264/56 (Miltenyi Biotec, Cat# 130–113-133; CD3^+^ T-cells), PE-CD270, Clone 122 (BioLegend, Cat# 318806; HVEM^+^), and APC-CD272, Clone mih26 (BioLegend, Cat# 344510; BTLA^+^). Proliferation was assessed on fixed lymphocytes from a subset of patient and control samples using Ki-67-PE-Vio770 (Miltenyi Biotec, Cat# 130–120-419; Ki67^+^). As above, Miltenyi MACSQuant was used to assess fluorescence, and data were analyzed with FlowJo version 9.3.2. Gating was again established with a fluorescence minus one approach and isotype control specimens were used to assess non-specific binding, as demonstrated in Figure 2.

Patient serum was isolated from whole blood samples using histopaque separation and centrifugation, and the supernatant was collected and stored at-80°C for utilization in cytokine arrays.

### 2.5. Cytokine arrays

Plasma samples were collected and stored as described above. Circulating cytokine analysis within plasma samples was undertaken according to manufacturer instructions using a commercially available cytokine bead array: LEGENDplex Mouse Inflammation Panel (13-plex) with a V-bottom plate (BioLegend, Cat# 552364). Serum samples from murine subjects were prepared per the manufacturer’s protocol.

This was repeated for patient serum samples, which were collected and stored as described above, using a LEGENDplex Human Inflammation Panel (13-plex) with a V bottom plate (BioLegend, Cat# 740808). Patient serum was prepared per the manufacturer’s protocol. All multiplex experiments were completed using MACSQuant Analyzer 10 (Miltenyi Biotec). LEGENDplex software suite (BioLegend) was used to analyze results.

### 2.6. Statistical analysis

When data were normally distributed, a *t*-test was used for S-CLP, Hem/CLP, and patient samples, and ANOVA for L-CLP samples. When data were not normally distributed in S-CLP, Hem/CLP, and patient populations, a Mann–Whitney *U*-test was applied. Results are presented as means ± standard error of the mean. Linear regression was used to correlate HVEM expression and APACHE II Score, and R2 was calculated. Statistical analysis was undertaken using GraphPad Prism version 8.4.3 for Windows (GraphPad Software, San Diego, CA, United States).^1^ Alpha was set to 0.05.

## 3. Results

### 3.1. Murine high-severity models failed to demonstrate significant changes in HVEM^+^BTLA^+^ co-expression

**Figure 3:**
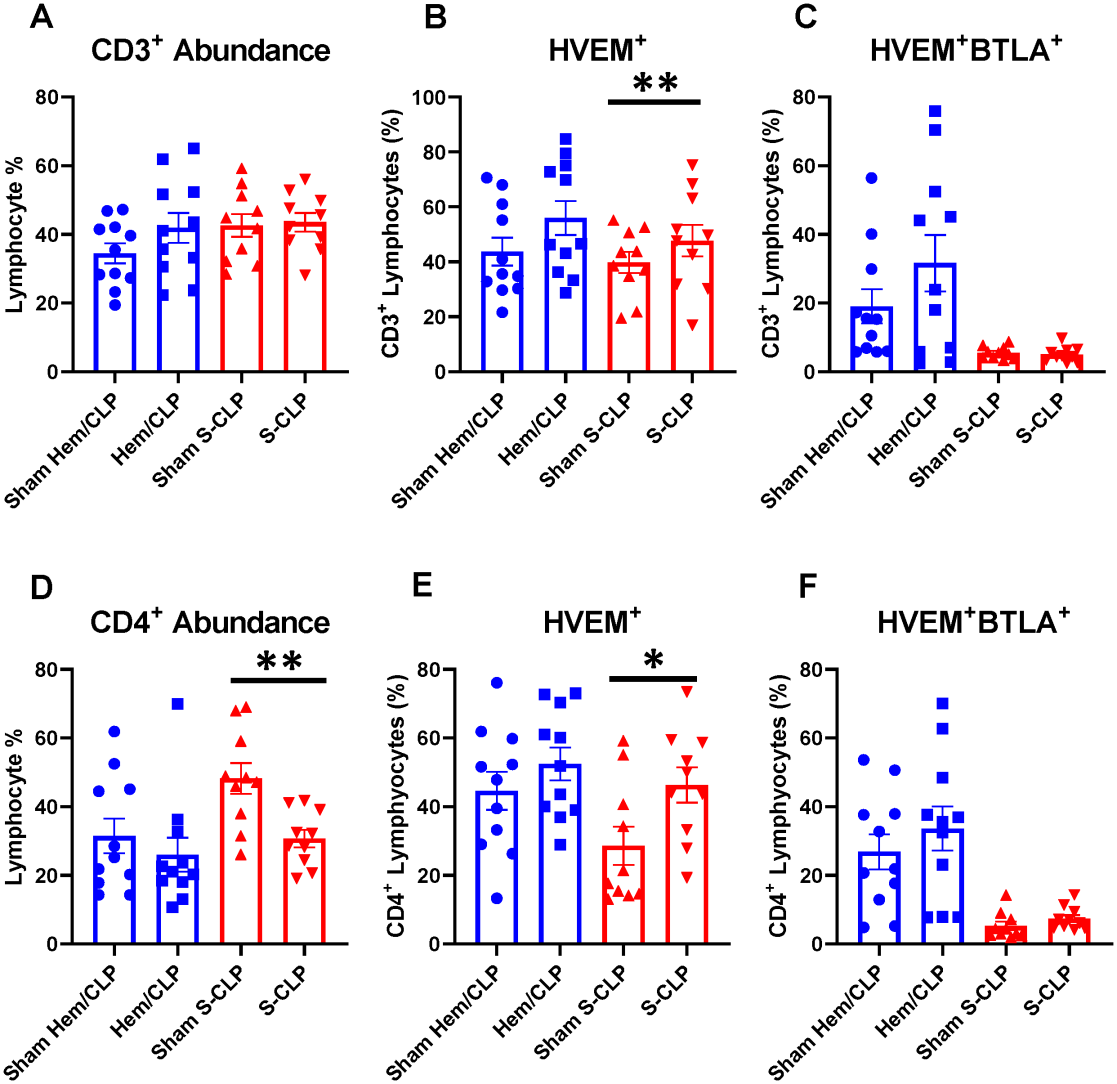
Thymic CD3^+^ and CD4^+^ lymphocytes demonstrated no change in HVEM^+^ BTLA^+^ co-expression after either Hem/CLP or S-CLP models, though CD3^+^ and CD4^+^ lymphocytes both demonstrated increased HVEM expression after S-CLP. **(A)** Thymic CD3^+^ lymphocyte abundance. **(B)** Thymic CD3^+^ lymphocyte HVEM^+^ expression. **(C)** Thymic CD3^+^ lymphocyte HVEM^+^ BTLA^+^ co-expression. **(D)** Thymic CD4^+^ lymphocyte abundance. **(E)** Thymic CD4^+^ HVEM^+^ expression. **(F)** Thymic CD4^+^ HVEM^+^ BTLA^+^ co-expression. **(A–F)** Summary graphs show mean ± SEM all after Hem/CLP and S-CLP treatments; [Sham Hem/CLP *n* = 11, Hem/CLP *n* = 11, Sham S-CLP *n* = 10, S-CLP *n* = 10]; significance *p < 0.05; **p < 0.01.

Indirect acute lung injury modeling (Hem/CLP) and severe CLP modeling were utilized to create high-severity injury profiles. Prior utilization of the Hem/CLP model in studies of indirect acute lung injury has produced approximately 45% mortality at the 48 h time point after treatment, with a total mortality of 54% in 7-day survival studies (25, 32). By comparison, the S-CLP model generated a 50% mortality at the 24 h time point, and the L-CLP model demonstrates a 27% mortality at 48 h and a 0% mortality at 24 h, the key time points explored in this study (33). In Hem/CLP thymus and spleen samples, data were noted to have significant heterogeneity (Supplementary Figures S1A–F), with no significant changes in the HVEM^+^ expression (Supplementary Figures S1B,E) or HVEM^+^BTLA^+^ co-expression (Supplementary Figures S1C,F) on any of the four investigated cell subtypes (CD3^+^, CD4^+^, CD8^+^, or B220^+^) in either immune compartment. When comparing the response in S-CLP mice to Hem/CLP mice, however, some notable trends emerge. Within the thymus, there was no change in CD3^+^ abundance (Figure 3A) or CD3^+^ HVEM^+^BTLA^+^ co-expression (Figure 3C) after either Hem/CLP or S-CLP; however, after S-CLP, there was an increase in HVEM^+^ expression on CD3^+^ lymphocytes (Figure 3B). Similarly, in thymic CD4^+^ cells, there remained an increase in HVEM^+^ expression after S-CLP (Figure 3E) despite a reduction in CD4^+^ lymphocyte abundance (Figure 3D); however, this did not correlate with a change in CD4^+^HVEM^+^BTLA^+^ co-expression (Figure 3F).

**Figure 4:**
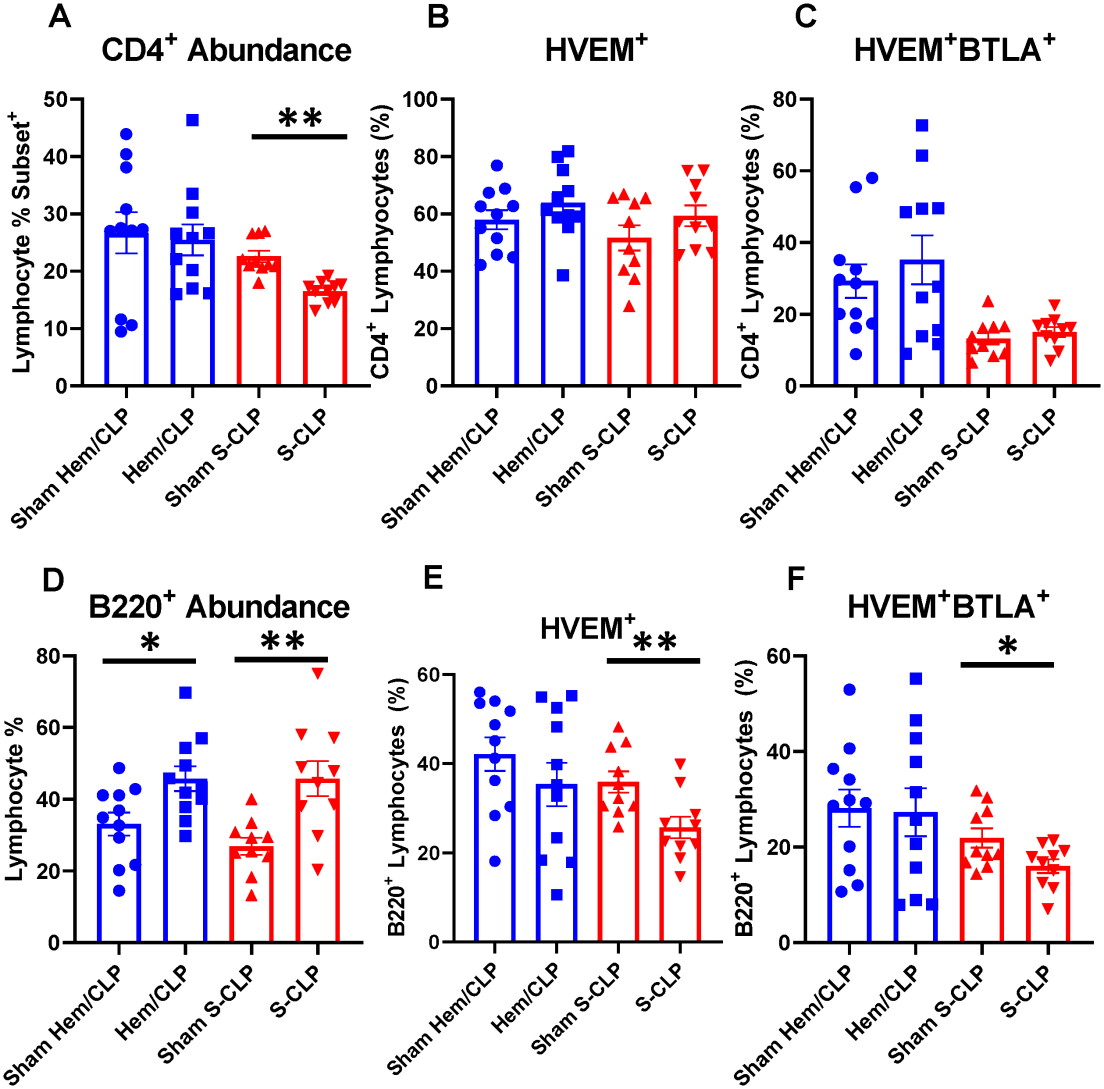
Splenic CD4^+^ demonstrated no change in HVEM^+^ or HVEM^+^ BTLA^+^ co-expression after either Hem/CLP or S-CLP, while splenic B220^+^ lymphocytes demonstrated increased abundance after both Hem/CLP and S-CLP, and reduced HVEM^+^ expression and HVEM^+^ BTLA^+^ co-expression after S-CLP. **(A)** Splenic CD4^+^ lymphocyte abundance. **(B)** Splenic CD4^+^ lymphocyte HVEM^+^ expression. **(C)** Splenic CD4^+^ lymphocyte HVEM^+^ BTLA^+^ co-expression. **(D)** Splenic B220^+^ lymphocyte abundance. **(E)** Splenic B220^+^ HVEM^+^ expression. **(F)** Splenic B220^+^ HVEM^+^ BTLA^+^ co-expression. **(A–F)** Summary graphs show mean ± SEM all after Hem/CLP and S-CLP treatments; [Sham Hem/CLP *n* = 11, Hem/CLP *n* = 11, Sham S-CLP *n* = 10, S-CLP *n* = 10]; significance *p < 0.05; **p < 0.01.

Within the spleen, there was a reduction in CD4^+^ lymphocyte abundance after S-CLP (Figure 4A), but no change in HVEM^+^ expression or HVEM^+^BTLA^+^ co-expression on CD4^+^ cells after either treatment (Figures 4B,C). B220^+^ lymphocytes demonstrated increased abundance in the spleen after both Hem/CLP and S-CLP treatments (Figure 4D). This was associated with a decrease in HVEM^+^ expression and a decrease in HVEM^+^BTLA^+^ co-expression after S-CLP treatment (Figures 4E,F). Notably, there was significant lymphocyte loss in both the thymus and the spleen after S-CLP treatment, with reductions in abundance of CD4^+^ and CD8^+^ lymphocytes in the thymus (Supplementary Figure S2A) and all four cell subsets in the spleen after S-CLP (Supplementary Figure S2D). This was associated with increased HVEM^+^ expression on thymic CD4^+^ and CD8^+^ lymphocytes (Supplementary Figure S2B), but no change in HVEM^+^BTLA^+^ co-expression on any subset in the thymus (Supplementary Figure S2C). One potential limitation of this exploration is that, given the profound severity of the S-CLP model and the mortality at the 24-h time point, co-expression changes in the survivors and non-survivors may drastically differ, but the characterization was only completed on the surviving mice. This warrants further exploration of the change in HVEM^+^BTLA^+^ co-expression at earlier time points in high-severity models to ensure emerging patterns that associate with eventual mortality are not missed.

### 3.2. Murine L-CLP modeling produced splenic and thymic HVEM^+^BTLA^+^ co-expression changes at the 48 h time point

**Figure 5:**
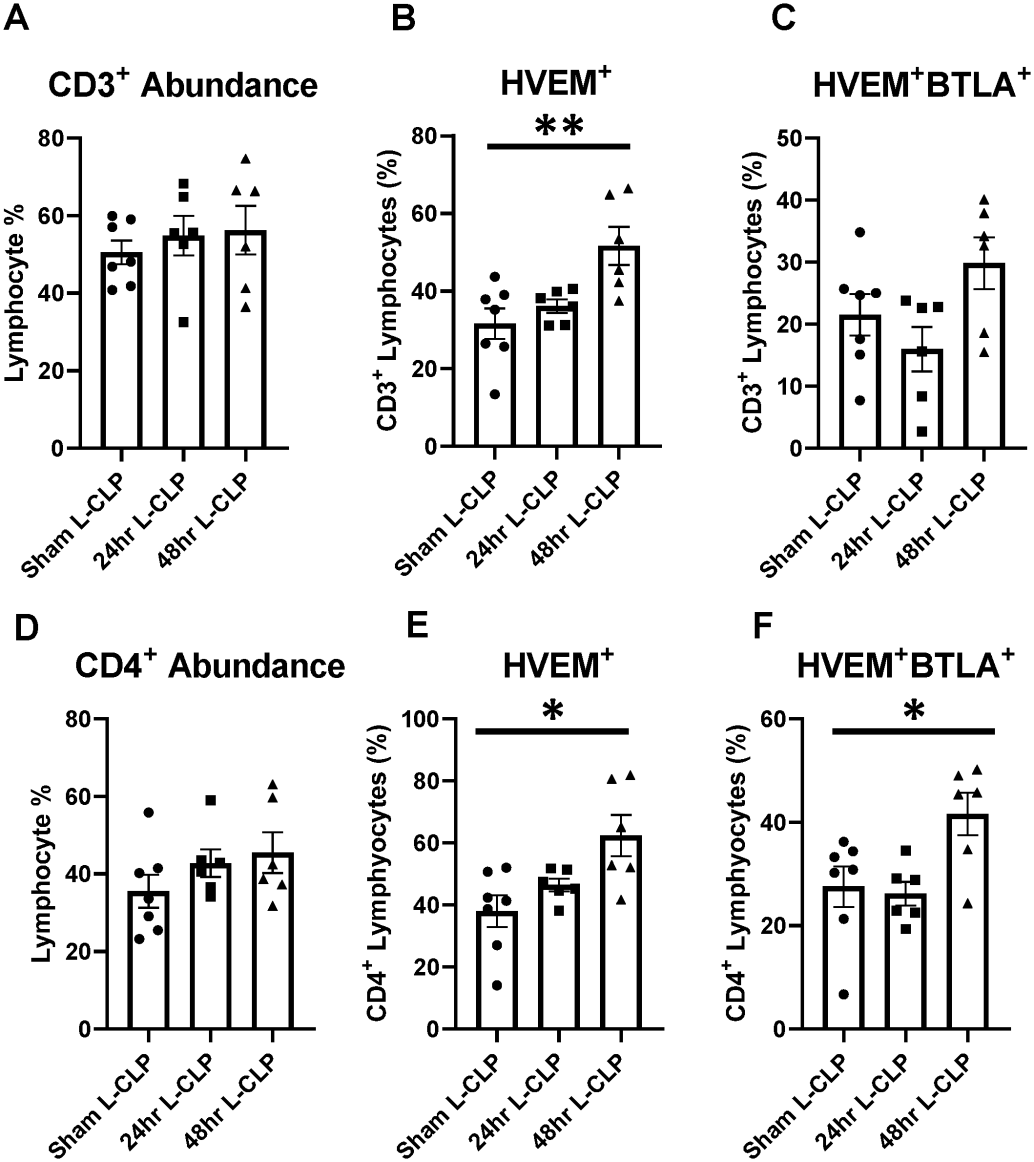
Thymic CD3^+^ and CD4^+^ lymphocytes both demonstrated increased HVEM expression after 48 h L-CLP, and thymic CD4^+^ lymphocytes also demonstrated a significant increase in HVEM^+^ BTLA^+^ co-expression at 48 h L-CLP. **(A)** Thymic CD3^+^ lymphocyte abundance. **(B)** Thymic CD3^+^ lymphocyte HVEM^+^ expression. **(C)** Thymic CD3^+^ lymphocyte HVEM^+^ BTLA^+^ co-expression. **(D)** Thymic CD4^+^ lymphocyte abundance. **(E)** Thymic CD4^+^ HVEM^+^ expression. **(F)** Thymic CD4^+^ HVEM^+^ BTLA^+^ co-expression. **(A–F)** Summary graphs show mean ± SEM all after L-CLP treatment; [Sham L-CLP *n* = 7, 24 h L-CLP *n* = 6, 48 h L-CLP *n* = 6]; significance *p < 0.05; **p < 0.01.

**Figure 6:**
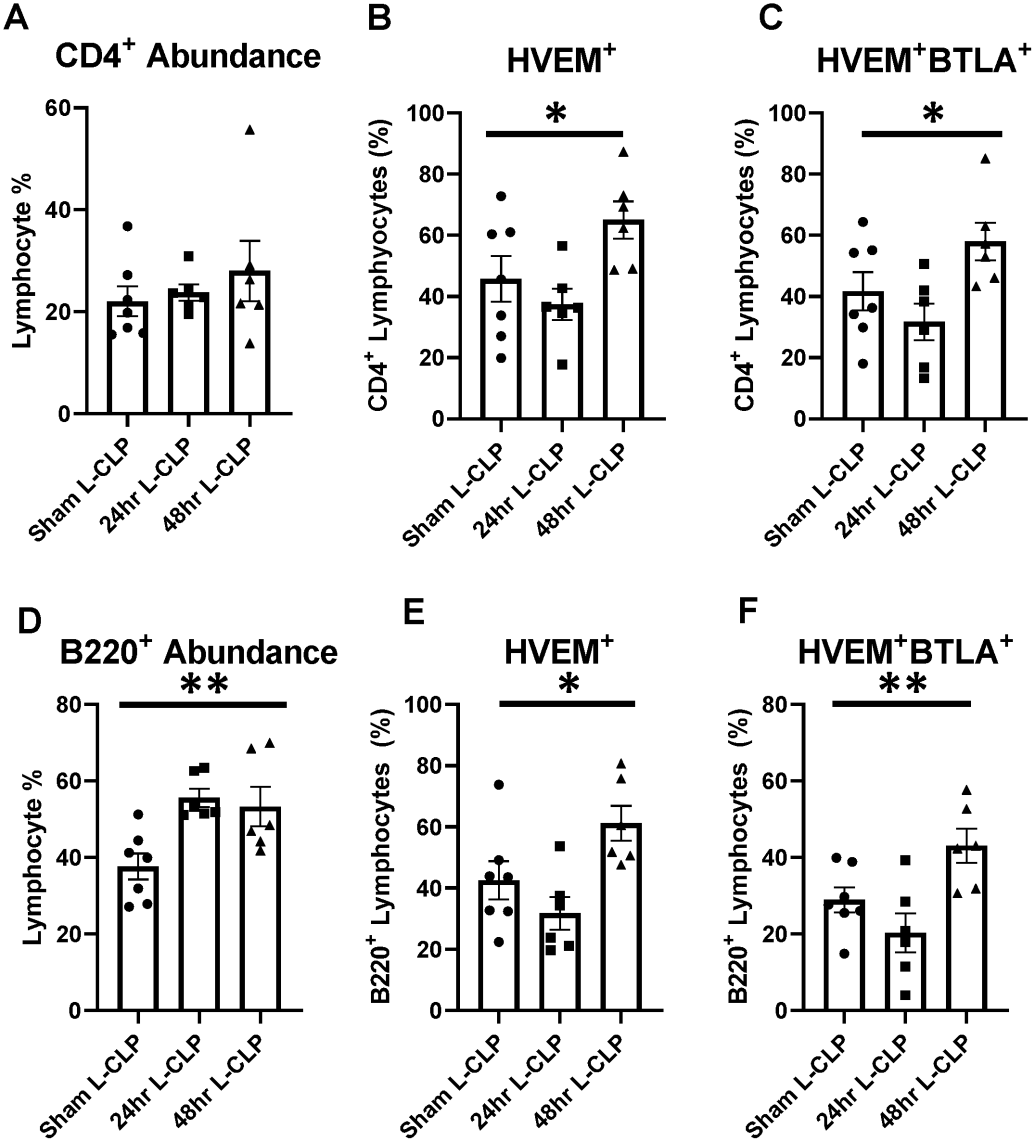
Splenic CD4^+^ lymphocytes demonstrated increased HVEM^+^ expression and HVEM^+^ BTLA^+^ co-expression after L-CLP, while splenic B220^+^ lymphocytes demonstrated increased abundance, and increased HVEM^+^ expression and HVEM^+^ BTLA^+^ co-expression after L-CLP. **(A)** Splenic CD4^+^ lymphocyte abundance. **(B)** Splenic CD4^+^ lymphocyte HVEM^+^ expression. **(C)** Splenic CD4^+^ lymphocyte HVEM^+^ BTLA^+^ co-expression. **(D)** Splenic B220^+^ lymphocyte abundance. **(E)** Splenic B220^+^ HVEM^+^ expression. **(F)** Splenic B220^+^ HVEM^+^ BTLA^+^ co-expression. **(A–F)** Summary graphs show mean ± SEM all after L-CLP treatment; [Sham L-CLP *n* = 7, 24 h L-CLP *n* = 6, 48 h L-CLP *n* = 6]; significance *p < 0.05; **p < 0.01.

To further explore the question of changes in HVEM^+^BTLA^+^ co-expression following critical illness, we needed to utilize a lower severity model that allowed consistent and appropriate survivorship at more delayed time points, prompting the introduction of the L-CLP treatment group, a model consistent with previously published CLP sepsis modeling (29, 30). This treatment only impacted the abundance of B220^+^ lymphocytes, both in the thymus and the spleen, without significant changes in CD3^+^, CD4^+^ (Figures 5A,D, 6A,D), or CD8^+^ lymphocyte abundance (Supplementary Figures S3A,D). Within the thymic compartment, however, L-CLP treatment induced increased HVEM^+^ expression on CD3^+^ and CD4^+^ lymphocytes (Figures 5B,E), and this was associated with a significant increase in HVEM^+^BTLA^+^ co-expression on CD4^+^ lymphocytes at the 48 h time point (Figure 5F), but no change in CD3^+^HVEM^+^BTLA^+^ co-expression (Figure 5C).

In the splenic compartment, L-CLP treatment was noted to induce no impact on CD4^+^ lymphocyte abundance (Figure 6A) but resulted in a significant increase in CD4^+^HVEM^+^ expression (Figure 6B) and increased CD4^+^HVEM^+^BTLA^+^ co-expression (Figure 6C). L-CLP treatment further induced an increase in splenic B220^+^ lymphocyte abundance (Figure 6D), with associated increases in both B220^+^HVEM^+^ expression (Figure 6E) and B220^+^HVEM^+^BTLA^+^ co-expression (Figure 6F) at the 48 h time point.

### 3.3. Multiple murine inflammatory cytokines increased at 24 h post-treatment from all treatment groups and returned to sham levels at 48 h post-L-CLP, commensurate with the timing of increases in HVEM^+^BTLA^+^ co-expression

**Figure 7:**
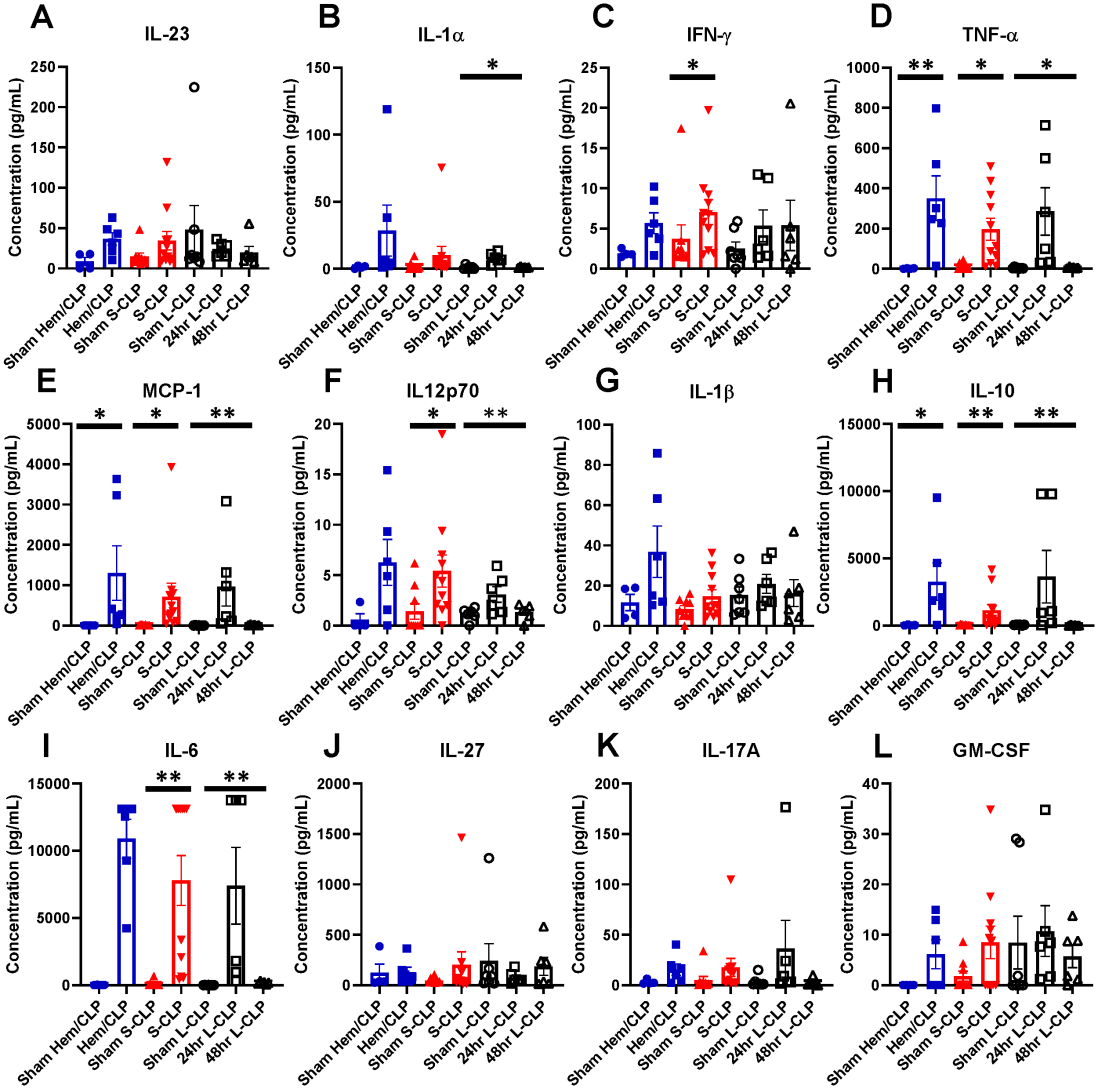
Mice demonstrate transient increases in multiple pro-inflammatory cytokines at the 24 h mark after all three models; however, this increase nearly always abates at the 48 h time point in L-CLP mice. **(A)** IL-23; **(B)** IL-1α; **(C)** IFN-γ; **(D)** TNF-α; **(E)** MCP-1; **(F)** IL12p70; **(G)** IL-1β; **(H)** IL-10; **(I)** IL-6; **(J)** IL-27; **(K)** IL-17A; **(L)** GM-CSF. **(A–L)** Summary graphs show mean ± SEM all after Hem/CLP, S-CLP, and L-CLP treatments [Sham Hem/CLP *n* = 11, Hem/CLP *n* = 11, Sham S-CLP *n* = 10, S-CLP *n* = 10, Sham L-CLP *n* = 7, 24 h L-CLP *n* = 6, 48 h L-CLP *n* = 6]; significance *p < 0.05; **p < 0.01.

To better understand the phenotype of critically ill mice with and without HVEM^+^BTLA^+^ co-expression changes, an inflammatory cytokine multiplex array was completed using samples from mice undergoing Hem/CLP, S-CLP, and L-CLP (Figures 7A–L). Changes in TNFα levels were noted in all three treatment groups; however, the increase noted after Hem/CLP, S-CLP, and L-CLP at 24 h was no longer present at the 48 h time point post-L-CLP (Figure 7D). This same pattern repeated with MCP-1 (Figure 7E), IL12p70 (Figure 7F), IL-10 (Figure 7H), and IL-6 (Figure 7I). IFN-γ did increase after S-CLP treatment at 24 h, but no changes were noted after Hem/CLP or L-LCP (Figure 7C). There was no significant change in IL-1β expression after any treatment (Figure 7G).

### 3.4. Critically ill patients demonstrate CD3^+^ lymphocyte loss, but residual CD3^+^ cells have increased HVEM^+^, BTLA^+^ expression, and HVEM^+^BTLA^+^ co-expression

We enrolled a total of 23 critically ill patients from the trauma and surgical ICUs at a single level-1 trauma center. Of the 23 patients, 13 (56.5%) sustained major traumatic injuries. Gender was not significantly different between patients and healthy controls, though healthy controls were significantly younger than patients. Moreover, 60.9% of patients had an active source of infection, 69.6% required mechanical ventilation, 35% were actively requiring vasopressor support at the time of the draw, and 22% required dialysis due to critical illness. The average APACHE II score for the total population was 19.1 (Table 1), and the average Injury Severity Score for the 12 traumatically injured patients was 26.4. Sources of infection included necrotizing soft tissue infections of the perineum and lower extremity skin and soft tissue, perforated hollow viscus injuries, and pneumonia. Approximately 69.6% of enrolled patients met systemic inflammatory response syndrome (SIRS) criteria, 43.5% met sepsis criteria, and 26.1% met septic shock criteria (31).

**Figure 8:**
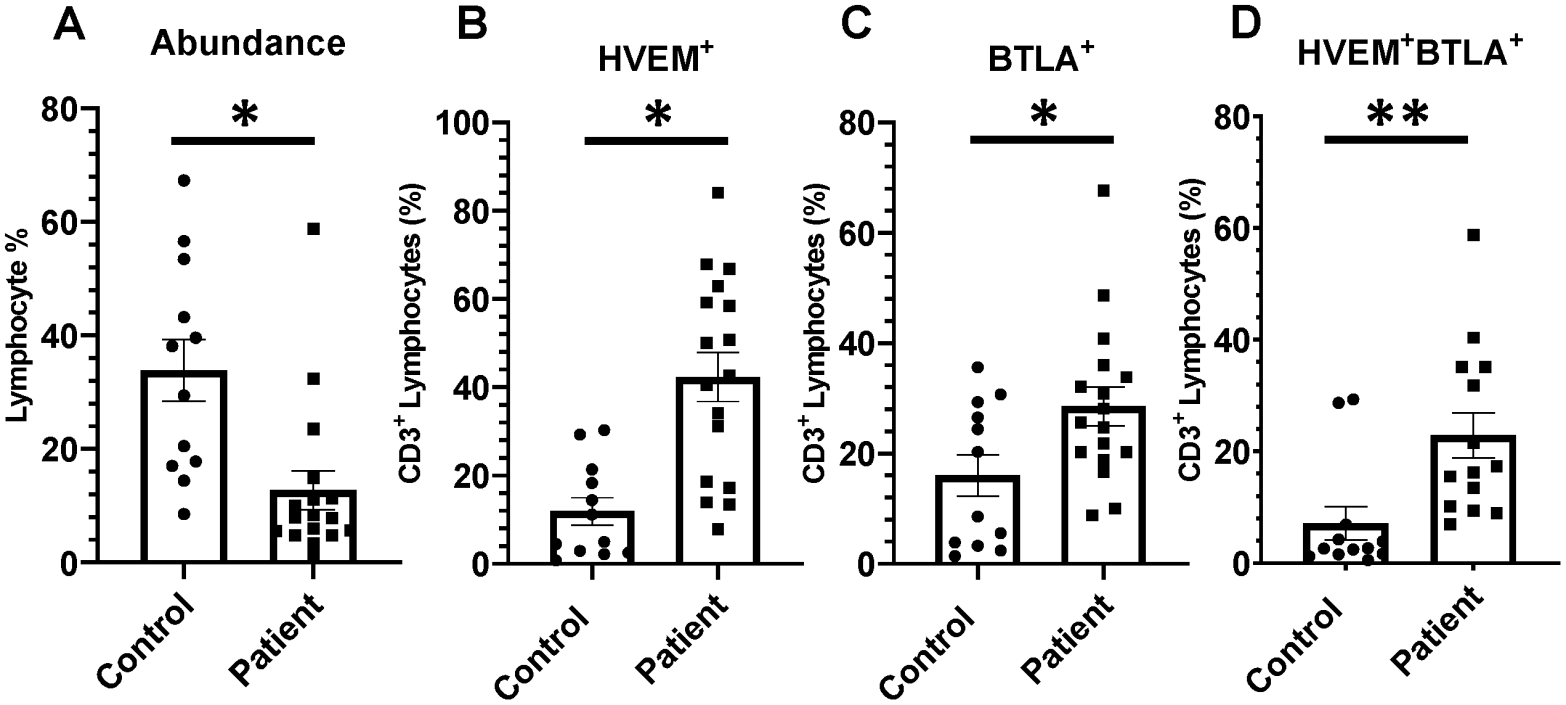
There was significant CD3^+^ lymphocyte loss after critical illness, with an associated increase in HVEM^+^ and BTLA^+^ expression on remaining CD3^+^ cells. There was also a significant increase in HVEM^+^BTLA^+^ co-expression on CD3^+^ cells after critical illness. **(A)** Circulating CD3^+^ lymphocyte abundance. **(B)** Circulating CD3^+^ lymphocyte HVEM^+^ expression. **(C)** Circulating CD3^+^ lymphocyte BTLA^+^ expression. **(D)** Circulating CD3^+^ HVEM^+^ BTLA^+^ co-expression. **(A–D)** Summary graphs show mean ± SEM all in critically ill patients as compared to healthy human controls [Control *n* = 12, Patients *n* = 17]; significance *p < 0.05; **p < 0.01.

**Figure 9:**
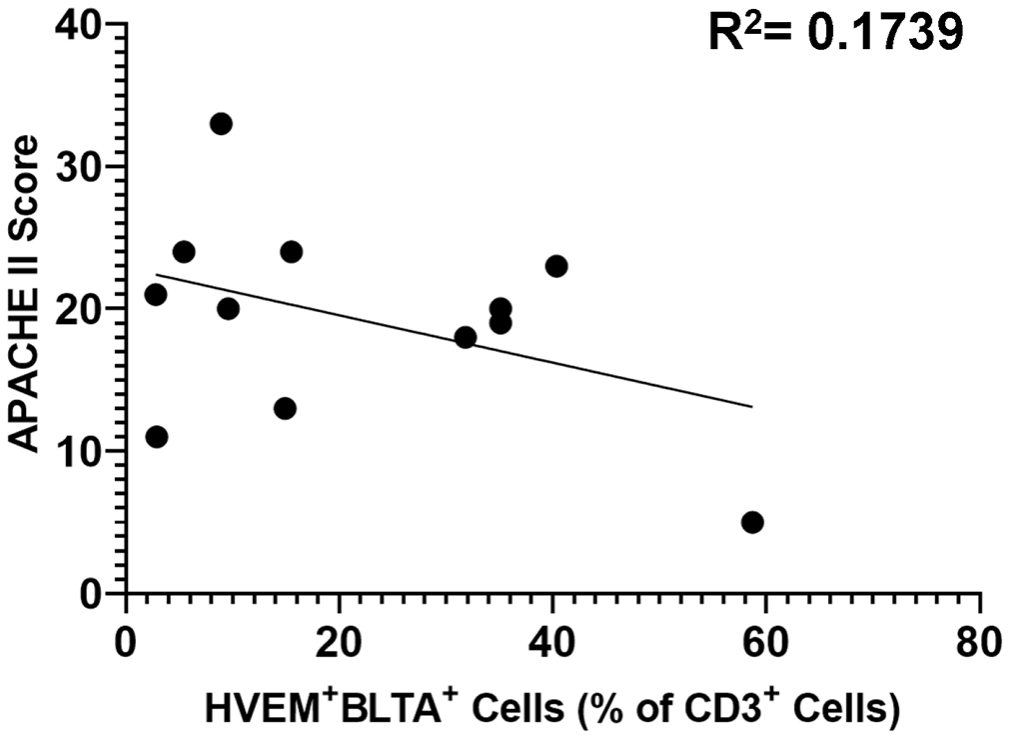
Correlation of patient APACHE II score with co-expression of HVEM^+^BTLA^+^ on CD3^+^ lymphocytes after critical illness. *R*^2^ = 0.1739.

Critically ill patients demonstrated CD3^+^ lymphocyte loss (Figure 8A) as compared to healthy controls. The remaining CD3^+^ lymphocyte population demonstrated increases in HVEM^+^ expression (Figure 8B), BTLA^+^ expression (Figure 8C), and HVEM^+^BTLA^+^ co-expression (Figure 8D). The relationship between patients’ APACHE II scores, a surrogate for disease severity in critically ill patients, and their CD3^+^ HVEM^+^BTLA^+^ co-expression demonstrated a negative correlation, with an *r*^2^ value of 0.1739 (Figure 9).

### 3.5. Critical illness altered patient CD3^+^ lymphocyte proliferation, and non-proliferating (Ki67^−^) lymphocyte expression profiles demonstrate increased co-expression compared to healthy controls

**Figure 10:**
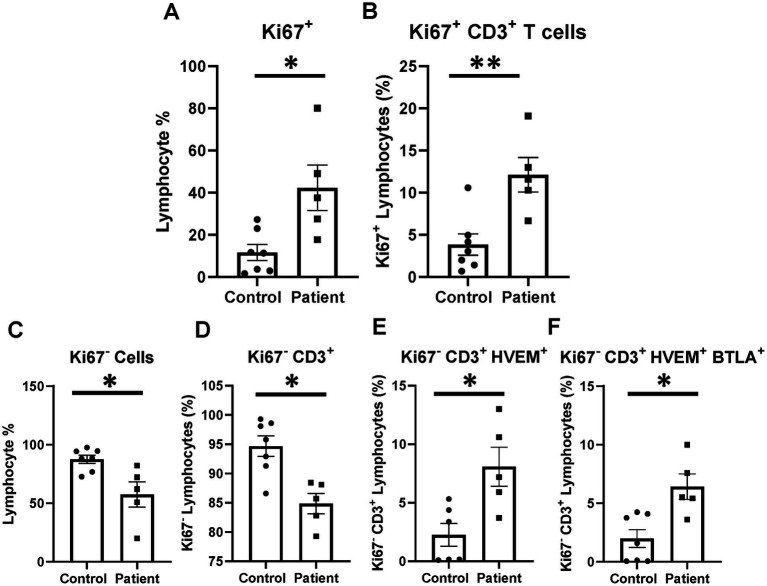
Lymphocyte proliferation after critical illness. There was a significant increase in total lymphocyte proliferation in critically ill patients, and this remained true in the CD3-positive subset. In the non-proliferating Ki67^−^ subset, there was CD3^+^ lymphocyte loss after critical illness, with an associated increase in HVEM^+^ expression and HVEM^+^BTLA^+^ co-expression remaining Ki67^−^CD3^+^ lymphocytes. **(A)** Circulating Ki67^+^ lymphocyte abundance. **(B)** Circulating Ki67^+^ CD3^+^ lymphocytes. **(C)** Circulating Ki67^−^ lymphocyte abundance. **(D)** Circulating Ki67^−^CD3^+^ lymphocytes. **(E)** Circulating Ki67^−^CD3^+^ lymphocytes HVEM^+^ expression. **(F)** Circulating Ki67^−^CD3^+^HVEM^+^ BTLA^+^ co-expression. **(A–F)** Summary graphs show mean ± SEM all in critically ill patients as compared to healthy human controls [Control *n* = 7, Patients *n* = 5]; significance *p < 0.05; **p < 0.01.

Proliferation activity was investigated for a subset of patients (*n* = 5) and healthy controls (*n* = 7) to better characterize the phenotype of elevated HVEM^+^BTLA^+^ co-expression after critical illness. Patients were noted to have higher Ki67^+^ lymphocytes than healthy controls, including higher levels of Ki67^+^CD3^+^ lymphocytes (Figures 10A,B). When exploring those non-proliferating cells, a reciprocal reduction in Ki67^−^ and Ki67^−^CD3^+^ lymphocytes was noted (Figures 10C,D). Of those remaining non-proliferating cells, there was significantly higher HVEM^+^ expression on Ki67^−^CD3^+^ cells in patients, with an associated increase in Ki67^−^CD3^+^HVEM^+^BTLA^+^ co-expression (Figures 10E,F).

### 3.6. Patient circulating cytokines demonstrated reduced levels of IFN-α2, TNF-α, IL-12p70, IL-17A, and IL-23 with an associated increase in IL-6 compared with healthy controls

**Figure 11:**
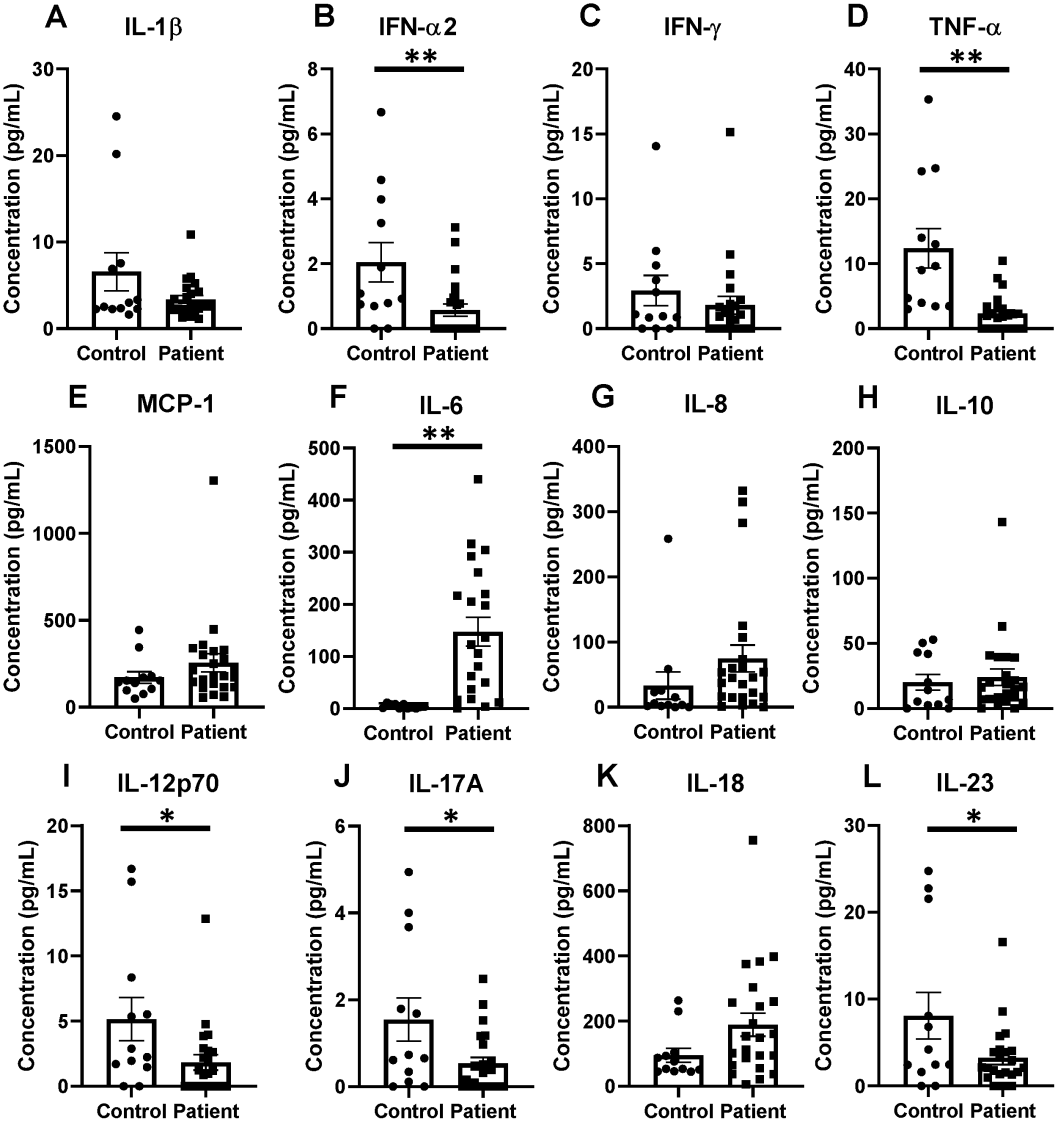
Inflammatory cytokines changes in critical illness, including notable decreases in TNF-α and increases in IL-6 when compared to healthy controls. **(A)** IL-1β. **(B)** IFN-α2. **(C)** IFN-γ. **(D)** TNF-α. **(E)** MCP-1. **(F)** IL-6. **(G)** IL-8. **(H)** IL-10. **(I)** IL-12p70. **(J)** IL-17A. **(K)** IL-18. **(L)** IL-23. **(A–L)** Summary graphs show mean ± SEM all in critically ill patients as compared to healthy human controls [Control *n =* 14, Patients *n* = 23]; significance *p < 0.05; **p < 0.01.

Patient serum cytokine results demonstrated a reduction in a number of inflammatory cytokines after critical illness (Figures 11A–L). Notably, there were significant reductions in levels of IFN-α2, TNF-α, IL-12p70, IL-17A, and IL-23 after critical illness (Figures 11B,D,F,I,J,L), but no change in levels of IL-1β, IFN-γ, MCP-1, IL-8, IL-10, and IL-18 (Figures 11A,C,E,G,K). In the explored panel of inflammatory cytokines, the only significant increase was observed in levels of IL-6, which was increased after critical illness compared to healthy controls (Figure 11F).

## 4. Discussion

Expression of HVEM and BTLA have previously been demonstrated to increase lymphocytes in both trauma- and sepsis-induced critical illness, with associated impact on overall septic mortality and changes in nosocomial infection risk (24, 26). More recent elucidation of HVEM’s ligand interactions has revealed a unique impact of HVEM/BTLA co-expression on downstream signaling of the HVEM pathway. HVEM/BTLA co-expression inhibits the typical downstream sequelae of HVEM ligand engagement, including preventing the activation of NFκB and release of inflammatory cytokines including TNF-α, IL-1β, IL-6, and IL-12p40 (17, 18). While circumstances, where this metaphorical break on immune regulation could be useful, are easy to imagine, presence of such a signaling blockade in the post-traumatic or post-septic response could limit the immune response to secondary insults. Therefore, HVEM/BTLA co-expression could provide a mechanistic, and potentially modifiable, explanation for the previously observed clinical outcomes in patients with altered lymphocyte HVEM and BTLA expression. This study utilized a variety of murine models of critical illness to explore how severity impacts the degree of HVEM^+^BTLA^+^ co-expression and associated downstream impacts, as well as patient-derived samples to better characterize the impact of HVEM^+^BTLA^+^ co-expression on lymphocyte proliferation and cytokine release.

### 4.1. In murine modeling, severity demonstrated no impact on HVEM^+^BTLA^+^ co-expression in the thymus or spleen; however, later time points in lower severity models impacted HVEM^+^BTLA^+^ co-expression

Modeling with Hem/CLP simulates a common complication of critical illness, both after trauma and sepsis indirect acute lung injury. Combining both intra-abdominal sepsis and hypotension, it clinically mirrors the stress of hypovolemic shock resulting from traumatic injury and hemorrhage, operative stress and anesthetic effect, or cardiogenic etiologies resulting in poor forward flow. In total, this model more robustly generates the end organ dysfunction, specifically within the lung, than other models, demonstrating significant changes to lung immune cell expression profiles, physiologic function, and cytokine milieu in prior studies (25, 32, 34). This model has previously been demonstrated to induce differential expression of HVEM in murine lung tissue, and septic-associated lung injury was noted to be mitigated by the administration of HVEM-blocking siRNA (19). The S-CLP model more than doubled the volume of ischemic bowel used in standard CLP modeling, in this experiment referred to as L-CLP, with complete cecal ligation, in an attempt to create a more advanced septic simulation. Given prior observations of differential HVEM^+^BTLA^+^ co-expression in critical illness, we expected these varying techniques to produce differing impacts on the degree of HVEM^+^BTLA^+^ co-expression and utilized a survey approach of multiple immune compartments to distinguish these differences.

Counter to our initial hypothesis, murine specimens treated with the higher severity models of critical illness exhibited minimal changes in HVEM^+^BTLA^+^ co-expression on lymphocytes. This pattern was largely consistent, including all CD3^+^, CD4^+^, and CD8^+^ lymphocytes from both tissues of origin. The greatest change in HVEM^+^BTLA^+^ co-expression was noted to occur in the lower severity model (L-CLP) at the late, 48 h time point. Co-expression increases were seen in L-CLP in both thymic and splenic CD4^+^ lymphocytes and in splenic B220^+^ lymphocytes. As readouts in this experiment were captured from murine immune compartments rather than circulating lymphocytes to facilitate consistent and strong cellular yields, this more delayed presentation of cells impacted by systemic disease is not surprising. Prior studies analyzing tissue injury characteristics of murine septic modeling have repeatedly demonstrated that lymphoid tissues are particularly susceptible to the steroid stress resulting from a septic challenge, demonstrating profound lymphoid organ apoptosis (35, 36). However, the time course of this ultimate lymphoid response is not immediate, and therefore the time points selected in the high-severity models may have been too early to fully appreciate this effect. Furthermore, characterization of thymic T lymphocyte behavior after sepsis has demonstrated that a great deal of the loss of early T lymphocyte progenitor cells in the thymus after sepsis is driven by inappropriate homing upon release from the bone marrow (37). This suggests that capturing T lymphocyte changes may be best appreciated in circulation. Finally, reduced splenic volume at the time of admission has been previously linked to higher severity of sepsis and worse outcomes in patients with pneumococcal infections. However, these findings were gathered in a patient cohort with pneumococcal bacteremia, a late sequela of pre-existing pneumococcal illness, again suggesting that these immune compartment changes may occur later in the disease course than assessed in this study (38).

### 4.2. Murine splenic B220^+^ lymphocytes demonstrated the most dynamic changes in abundance, HVEM^+^ expression and HVEM^+^BTLA^+^ co-expression following all levels of simulated critical illness

In exploring the HVEM^+^BTLA^+^ co-expression on cells from the two immune compartments, thymus and spleen, and the four total cell types, CD3^+^, CD4^+^, CD8^+^, and B220^+^ lymphocytes, the only cell subset to demonstrate consistent changes in HVEM^+^BTLA^+^ co-expression after critical illness were the splenic B220^+^ lymphocytes. Splenic B220^+^ lymphocytes significantly increased in abundance after all three treatments were applied, and HVEM^+^ expression increased on B220^+^ cells after 48 h in L-CLP-treated mice, but significantly declined at 24 h after S-CLP treatment. Similarly, HVEM^+^BTLA^+^ co-expression significantly increased on splenic B220^+^ lymphocytes in L-CLP samples at 48 h, but significantly declined at 24 h after S-CLP and did not change after Hem/CLP. This temporal pattern in changing expression patterns on splenic B220^+^ cells suggests that they are a more heavily HVEM-regulated cell subset in the time following critical illness. The increasing B220^+^ lymphocyte abundance preferentially expresses HVEM^+^ with BTLA^+^ over time, selecting for and expanding this population of more inert cells. This must be interpreted in the context of the known profound lymphocyte loss that occurs after septic and traumatic challenges in mice and patients. Therefore, these changes represent altered frequencies and disproportionate expression patterns within the small residual population of lymphocytes.

B220^+^ lymphocytes are essential to generating lasting responses to non-self-blood-born antigens. Specifically within the splenic compartment, B220^+^ cells are selected for maturation from larger populations of precursors that migrate from the bone marrow through an active process that evaluates qualitative and quantitative signals derived from the B cell receptors (39). Infectious responses within the spleen have been demonstrated to produce lymphoid or stromal hyperplasia, lymphoid atrophy, and disorganization of splenic compartments (40, 41). As lymphoid organs such as the spleen are configured to allow cellular interaction and the subsequent proliferation and maturation that is necessary to promote responses to immunological challenges, this post-infectious compartmental disorganization compounded by increasing numbers of B220^+^ cells with higher levels of HVEM^+^BTLA^+^ co-expression, and lack of normal informative signaling, produces a profoundly atypical response to new insults encountered during this time.

### 4.3. Critically Ill patients demonstrate significantly higher CD3^+^HVEM^+^BTLA^+^ co-expression than healthy controls; however, increased co-expression did not correlate positively with increased illness severity graded by APACHE II score

In keeping with prior observations on critically ill septic patients, we demonstrate CD3^+^ lymphocyte loss, and increased HVEM expression on remaining CD3^+^ cells after critical illness in patients (26). Unique to prior data, we demonstrate that the increased HVEM^+^ expression on CD3^+^ lymphocytes comes in the form of significantly higher co-expression of HVEM^+^BTLA^+^, suggesting that this increased expression may not result in an increased immune informing signal as previously thought. Given that HVEM^+^BTLA^+^ co-expression results in an inert signaling complex, where HVEM is unable to instruct immune cells on the most appropriate response, this represents a plausible mechanism contributing to the immune suppression of critical illness seen frequently in patients after sepsis and trauma (16, 18). Furthermore, given the general absence of HVEM^+^BTLA^+^ co-expression changes seen in multiple murine models of critical illness when nearly 35% of circulating human lymphocytes expressed this pattern, this finding represents a clear difference in animal modeling and patient responses which might begin to explain the differential responses to checkpoint regulator blockade therapy that have been seen in patients as compared to mice in recent clinical trials (4, 42).

Contrary to murine modeling, establishing the time point for the onset of an infectious insult, and even a traumatic injury pattern, is difficult and introduces significant heterogeneity into the temporal component of this clinical data. While patients were enrolled within 48 h of ICU admission after trauma, or within 48 h of presentation of sepsis, variables such as delayed presentation and prolonged on-scene extrication times can alter this timeline. One measure of the severity of critical illness that has been frequently utilized is the APACHE II score. APACHE II score has been demonstrated to predict mortality and outcomes in intensive care units housing surgical patients, and scores calculated later in hospital courses have been previously demonstrated to even improve the predictive value of this score (43, 44). We utilized APACHE II scores for our mixed population of critically ill patients as its efficacy has been demonstrated both after trauma and sepsis in the past. In this study, counter to our original hypothesis, the APACHE II score was not found to be positively associated with the degree of HVEM^+^BTLA^+^ co-expression after critical illness, with an *r*^2^ value of 0.1739. This further substantiates the findings in the murine component of this study that, while HVEM^+^BTLA^+^ co-expression is induced by critical illness, the degree is unrelated to the severity of critical illness. It should be noted that factors such as age, as well as pre-existing medical co-morbidities, do influence the APACHE II score, and the impact of these could not be controlled for in analyzing this data. This patient population does represent a heterogeneous group; however, they are unified through their requirement for intensive care for major septic or traumatic insults, and all are at risk of post-critical illness secondary infections. Given their similar risk profiles for secondary infections, this unifying trend of CD3^+^ lymphocyte HVEM^+^BTLA^+^ co-expression suggests a promising immune modulation target warranting further exploration.

### 4.4. Critical illness induced an increase in proliferating CD3^+^ lymphocytes; however, non-proliferating CD3^+^ cells demonstrated increased HVEM^+^BTLA^+^ co-expression suggesting cells not appropriately responding to the stimulus of critical illness may be blocked by such co-expression

Following the septic challenge, immune cells including lymphocytes have been demonstrated to increase proliferation as they are stimulated to expand and address inciting pathogens. As expected, there was an increase in the number of proliferating cells (Ki67^+^) in critically ill patients, reflecting an expected mounting immune response. This remained true when the CD3^+^ lymphocyte subset was examined. However, when exploring the expression profiles of non-proliferating cells, we found that critically ill patients were more likely to co-express HVEM^+^BTLA^+^ on CD3^+^Ki67^−^ lymphocytes than healthy controls. Mechanistically, with co-expression’s prevention and HVEM’s ability to inform and direct immune responses, the finding of higher HVEM^+^BTLA^+^ co-expression on the non-proliferating (Ki67^−^) lymphocyte subset supports that observed co-expression results in active alterations in immune proliferation *in vivo*.

### 4.5. After critical illness, patients demonstrated suppression of a variety of pro-inflammatory cytokines despite a high rate of ongoing active infections with isolated increases in IL-6 levels

In the patient’s inflammatory cytokine profile, a number of significantly reduced levels of proinflammatory mediators stood out as compared to healthy controls. We observed significant reductions in levels of TNF-α, IFN-α2, IL-12p70, IL-17A, and IL-23 after critical illness. The reduction in TNF-α interestingly differs from the murine findings of an increase at the 24-h time point after Hem/CLP, S-CLP, and L-CLP, but a return to sham levels at 48 h post-injury. Given that TNF-α is a direct downstream signal of the HVEM pathway, its reduction in patients suggests an impact from the notable increases in HVEM^+^BTLA^+^ co-expression observed in these samples.

No changes were observed in levels of IL-1β, IFN-γ, MCP-1, IL-8, IL-10, and IL-18 when compared to healthy controls; however, the strongly anti-inflammatory IL-6 was the only cytokine noted to increase significantly in patients after critical illness. This again differs slightly from our murine findings, where IL-6 was noted to increase at the 24 h time point in both CLP models but returned to sham levels at the 48 h time point. Multiple prior studies have sought to describe the cytokine milieu associated with immunosuppression following chronic critical illness, with consistently noted increased IL-6 levels, as seen in both the murine and patient populations in this study, as well as increased IL-10, again seen in the murine results (45, 46). Furthermore, as mentioned above, the time a septic or traumatic insult begins in a patient is much more ambiguous than in murine modeling, given that, this data more likely represent later time points than 48 h after initiation of critical illness in many cases. In total, these findings suggest that the downstream cytokine changes resulting from critical illness and associated with simultaneous changes in lymphocyte HVEM^+^BTLA^+^ co-expression are durable and result in a generally anti-inflammatory state, providing a more hospitable environment for secondary infections. This supports prior publications where demonstrated increases in nosocomial infections were associated with elevated BTLA and HVEM expressions in critically ill patients (23, 24, 26).

### 4.6. Future directions

This study demonstrated consistent patterns in HVEM^+^BTLA^+^ co-expression on peripheral blood lymphocytes after sepsis- and trauma-induced critical illness with associated lymphocyte proliferation and cytokine changes that suggest a potential mechanistic explanation for a component of critical illness-induced immunosuppression. However, our investigation into the factors that impact the degree of HVEM^+^BTLA^+^ co-expression produced by a particular injury pattern failed to expose a consistent predictor of this phenotype. Further investigation of HVEM^+^BTLA^+^ co-expression both in the circulating blood as well as various immune compartments at later time points after septic challenge in murine models provides a promising opportunity to better characterize this interesting immune-modulating phenomenon. Additionally, the exploration of exposure of critically ill mice to secondary infectious events and the correlation of their response to these infections with their HVEM^+^BTLA^+^ co-expression may further expand our understanding of the impact co-expression levels have on critical illness-induced immunosuppression.

Regarding human subjects, exploring samples from additional patients drawn at multiple time points throughout their critical illness additionally provides an opportunity to better explore the phenotype of HVEM^+^BTLA^+^ co-expression over time and correlate it to the potential onset of secondary infections. Finally, future studies of HVEM^+^BTLA^+^ co-expression should explore the function of these patient co-expressing lymphocytes *ex-vivo* as compared to the small volume of healthy control patient co-expressing lymphocytes. As the ability to clinically evaluate patients’ more individualized cellular phenotypes in critical illness continues to expand, HVEM^+^BTLA^+^ co-expression should be considered as a marker of risk for infection as well as a potential therapeutic target to modulate the immune response to critical illness, offering a novel means of preventing critical illness-related complications.

## Data availability statement

The original contributions presented in the study are included at: https://doi.org/10.26300/pb48‐rh89. Further inquiries can be directed to the corresponding author.

## Ethics statement

The studies involving human participants were reviewed and approved by the Institutional Review Board at Rhode Island Hospital (IRB study #413013). The patients/participants provided their written informed consent to participate in this study. The animal study was reviewed and approved by The Animal Use Committee of Rhode Island Hospital (AWC# 5064–18 and 5,054–21).

## Author contributions

BA and ET completed S-CLP, L-CLP, and Hem/CLP animal experiments and tissue processing. MW and DH participated in human subject enrollment, specimen collection, and processing. CG, MW, ET, and BA completed flow cytometric analysis. MW completed the cytokine analysis. C-SC and AA contributed to conclusion and discussion of the manuscript. All authors contributed equally to the study design and were involved in the manuscript assembly.

## Funding

This work was supported by the National Institutes of Health [grant numbers R35 GM118097 (AA), R25 GM083270 (CG), K08-GM110495 (DH)] and the Armand D. Versaci Research Scholar in Surgical Sciences Fellowship (MW) as well as a Post/Pre-doctoral fellowship awards from the National Institutes of Health [grant number T32 GM065085 (MW and ET) and T32 HL134625 (BA and CG)].

## Conflict of interest

The authors declare that the research was conducted in the absence of any commercial or financial relationships that could be construed as a potential conflict of interest.

## Publisher’s note

All claims expressed in this article are solely those of the authors and do not necessarily represent those of their affiliated organizations, or those of the publisher, the editors and the reviewers. Any product that may be evaluated in this article, or claim that may be made by its manufacturer, is not guaranteed or endorsed by the publisher.

## Footnotes

^1^ www.graphpad.com
