# Supplementary material for: Lymphocyte HVEM/BTLA co-expression after critical illness demonstrates severity indiscriminate upregulation, impacting critical illness-induced immunosuppression
Source: Front Med (Lausanne). 2023 May 25;10:1176602. doi: 10.3389/fmed.2023.1176602 (PMC10248445; doi:10.3389/fmed.2023.1176602)
Supplement: Supplementary file 1 [file Data_Sheet_1.docx]

Supplementary Material

- **Lymphocyte** **HVEM/BTLA Co-Expression After Critical Illness Demonstrates Severity Indiscriminate Upregulation, Impacting Critical Illness Induced Immunosuppression.**

**Michelle E. Wakeley, MD; Brandon E. Armstead, MS^,2^; Chyna C. Gray, BS; Elizabeth W. Tindal, MD, MPH; Daithi S. Heffernan, MD; Chun-Shiang Chung, PhD; and Alfred Ayala, PhD^*^**

*** Correspondence:** Alfred Ayala, PhD: aayala@lifespan.org

##
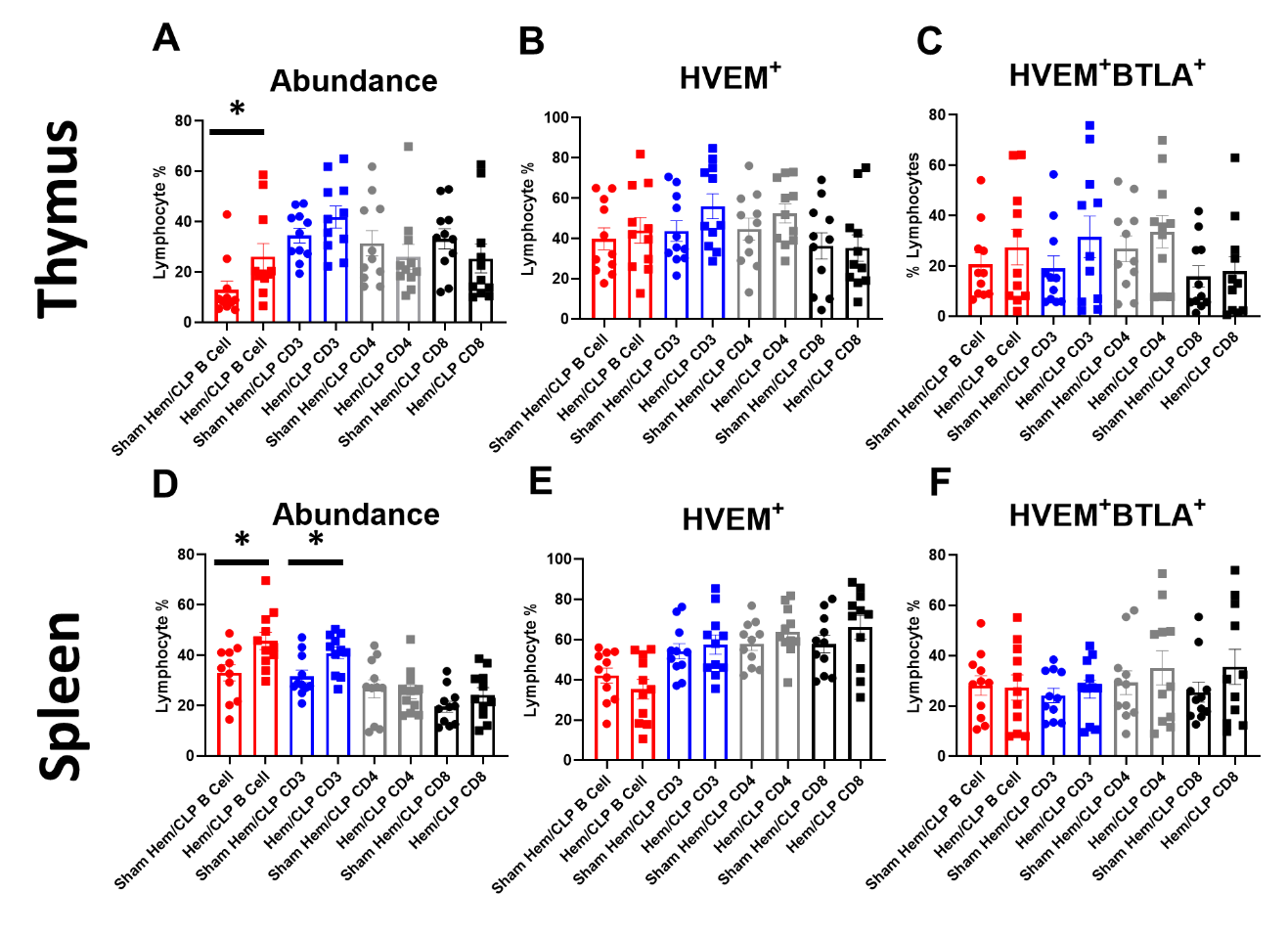
Supplementary Figures

**Supplementary Figure 1.** The figure legends are required to have the same font as the main text, 12 point normal Times New Roman, single spaced. Please use a single paragraph for each legend and prepare the figures keeping in mind the PDF layout.

**
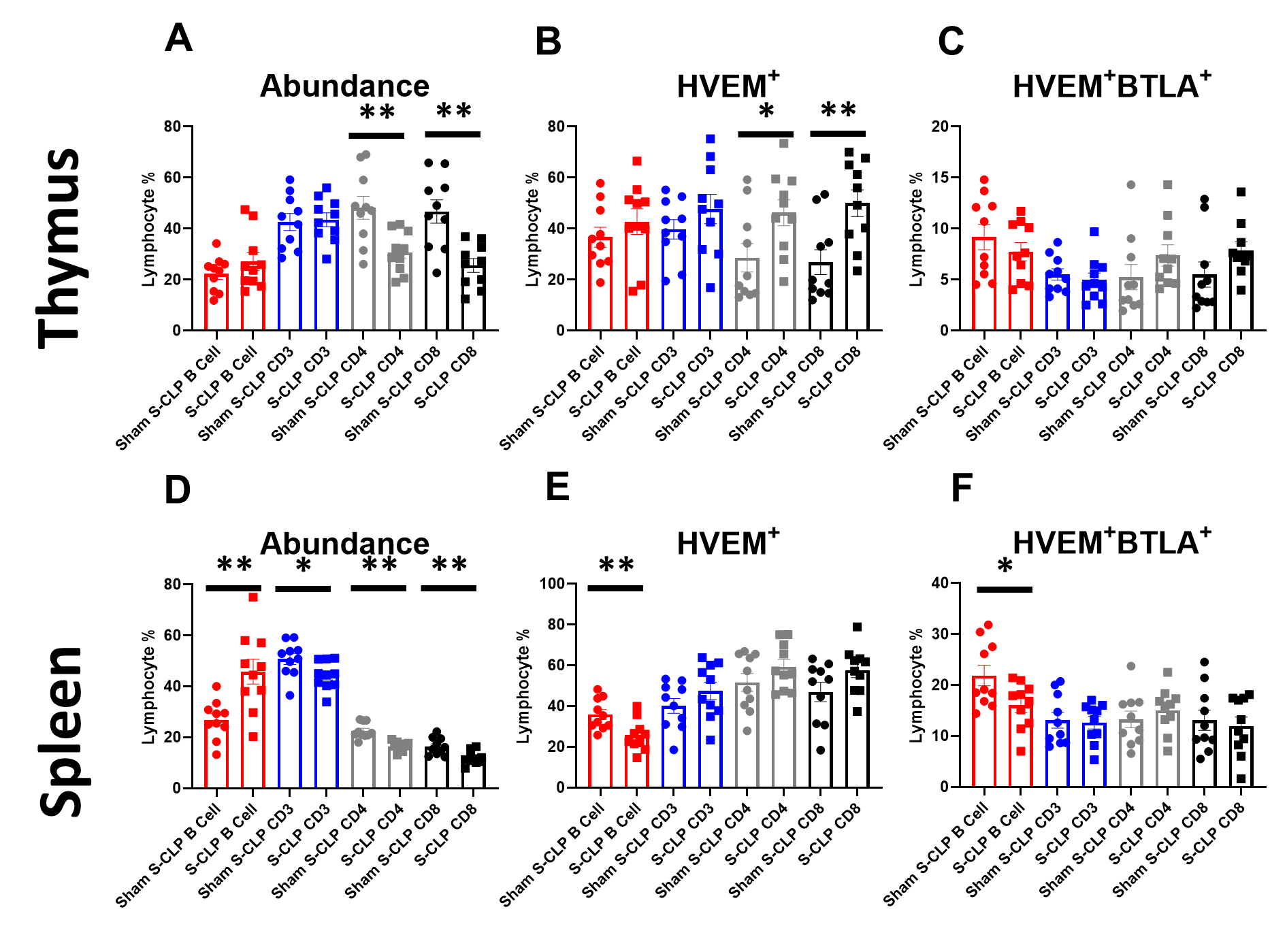
**

**Supplemental Figure 2. S-CLP Composite Data.** S-CLP composite data for all cell types test (B220^+^, CD3^+^, CD4^+^ and CD8^+^ thymic abundance (a), HVEM^+^ expression (b) and HVEM^+^BTLA^+^ co-expression (c), and splenic abundance (d), HVEM^+^ expression (e) and HVEM^+^BTLA^+^ co-expression (f). * p<0.05; ** p<0.01.


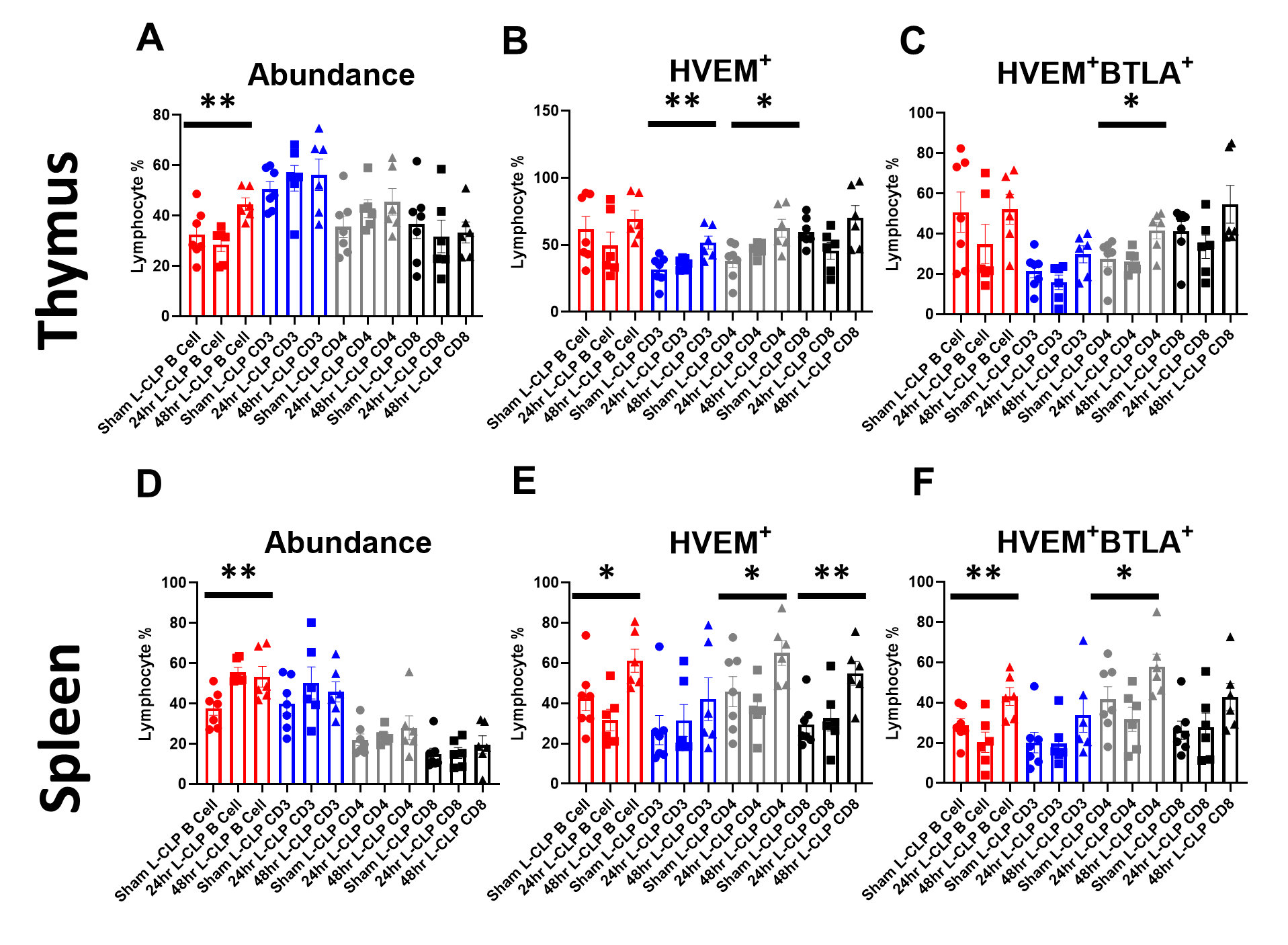


**Supplemental Figure 3. L-CLP Composite Data.** L-CLP composite data for all cell types test (B220^+^, CD3^+^, CD4^+^ and CD8^+^ thymic abundance (a), HVEM^+^ expression (b) and HVEM^+^BTLA^+^ co-expression (c), and splenic abundance (d), HVEM^+^ expression (e) and HVEM^+^BTLA^+^ co-expression (f). * p<0.05; ** p<0.01.
